# Urbanization Impacts the Physicochemical Characteristics and Abundance of Fecal Markers and Bacterial Pathogens in Surface Water

**DOI:** 10.3390/ijerph16101739

**Published:** 2019-05-16

**Authors:** Tianma Yuan, Kiran Kumar Vadde, Jonathan D. Tonkin, Jianjun Wang, Jing Lu, Zimeng Zhang, Yixin Zhang, Alan J. McCarthy, Raju Sekar

**Affiliations:** 1 Department of Biological Sciences, Xi’an Jiaotong-Liverpool University, Suzhou 215123, China; Tianma.Yuan@xjtlu.edu.cn (T.Y.); Kumar.Kiran@xjtlu.edu.cn (K.K.V.); 2 School of Biological Sciences, University of Canterbury, Christchurch 8140, New Zealand; jdtonkin@gmail.com; 3 Nanjing Institute of Geography and Limnology, Chinese Academy of Sciences, Nanjing 210008, China; jjwang@niglas.ac.cn; 4 Futurepolis LLC, Suzhou 215021, China; jinglu.lyu@gmail.com; 5 Institute of Integrative Biology, University of Liverpool, Liverpool L69 3BX, UK; zimeng.zhang@liverpool.ac.uk; 6 Department of Health and Environmental Sciences, Xi’an Jiaotong-Liverpool University, Suzhou 215123, China; Yixin.Zhang@xjtlu.edu.cn; 7 Microbiology Research Group, Institute of Integrative Biology, University of Liverpool, Liverpool L69 7ZB, UK; aj55m@liverpool.ac.uk

**Keywords:** urbanization, water quality, nutrients, microbial contaminations, fecal markers, pathogens, Suzhou canals

## Abstract

Urbanization is increasing worldwide and is happening at a rapid rate in China in line with economic development. Urbanization can lead to major changes in freshwater environments through multiple chemical and microbial contaminants. We assessed the impact of urbanization on physicochemical characteristics and microbial loading in canals in Suzhou, a city that has experienced rapid urbanization in recent decades. Nine sampling locations covering three urban intensity classes (high, medium and low) in Suzhou were selected for field studies and three locations in Huangshan (natural reserve) were included as pristine control locations. Water samples were collected for physicochemical, microbiological and molecular analyses. Compared to medium and low urbanization sites, there were statistically significant higher levels of nutrients and total and thermotolerant coliforms (or fecal coliforms) in highly urbanized locations. The effect of urbanization was also apparent in the abundances of human-associated fecal markers and bacterial pathogens in water samples from highly urbanized locations. These results correlated well with land use types and anthropogenic activities at the sampling sites. The overall results indicate that urbanization negatively impacts water quality, providing high levels of nutrients and a microbial load that includes fecal markers and pathogens.

## 1. Introduction

Urbanization is occurring globally and increasing at an extremely rapid rate in most developing countries, particularly in Asia and Africa [1]. Currently, approximately 55% of the world’s population lives in urban areas, which is projected to increase to 68% by 2050 [1]. The increase in urbanization is expected to be especially apparent in three countries—India, China and Nigeria, which will together account for 35% of the total increase in urban global population. In China, at the end of 2017, 58.5% of the population lived in urban areas [2] and it has been estimated that by 2030, the middle-class population resident in the cities could be up to one billion, corresponding to 70% of China’s projected total population [3]. Although rapid urbanization has created wealth and improved social conditions and human well-being [4], it has also created many problems, such as pressure on resource scarcity and environmental pollution [5,6]. Overcoming the negative environmental impacts of rapid urbanization is becoming one of the major themes of environmental studies in China, particularly water pollution [7].

Freshwater ecosystems are affected by both non-point and point source pollution, which leads to eutrophication, excessive algal growth, and chemical and microbial contaminations [8]. The decline in water quality has been a major problem in China in the past few decades despite significant progress having been made to protect surface water quality [9]. Notable pollutants in China include industrial effluent, domestic sewage and agricultural run-off [10]. Urbanization has been reported to affect the water quality of city rivers with excess nutrients, endocrine disrupting chemicals, heavy metals, antibiotics and steroid hormones [11], particularly in developed regions and large cities with increased human activity [12]. Urbanization density has the potential for predicting water quality [13] and urbanization has been positively related to multiple nutrients (total nitrogen and total phosphorus) and indicator bacteria (thermotolerant coliforms) [14]. In Beijing—the capital of China—landscape characteristics are significantly correlated with water quality in watersheds with high urban intensification [15], and rapid urbanization with intense land use, land cover change and population growth have a great impact on physicochemical variables [16]. Combined, these pressures threaten the safety of drinking water supplies and sustainability in the region [17].

Monitoring of urban waters is required to address the issue of sewage-associated pathogens and to implement remedial actions. Traditionally, culture-based methods such as fecal indicator bacteria (FIB) enumeration were commonly used for monitoring of fecal pollution to address the associated human health risk [18]. However, FIB enumeration to monitor the microbial quality of environmental waters has several limitations. For instance, these bacteria may persist and multiply outside of the host gastrointestinal tract, leading to difficulty in predicting recent fecal contamination in surface waters [19], and the correlation between FIB and pathogen presence is poor [20]. The main limitation with FIB is that it cannot identify the origin or source of fecal contamination [21], which is mandatory to depict the human health risk and implement remedial actions [22]. Therefore, microbial source tracking (MST) techniques have been developed to identify the origin of fecal sources [23]. Lakes, rivers, and canals situated in high population density urban areas provide sites for recreational activities and may pose a significant public health risk due to microbial pathogens found in these waters [24]. Evaluating the occurrence of bacterial pathogens in water bodies, particularly in high urbanization areas, is therefore vital to understand the environmental impact of urbanization [25].

The aim of this study was to assess the physicochemical and microbiological parameters (fecal markers and pathogens) in the Suzhou canals across a gradient of urban intensification. Suzhou is the second largest city in the South-Eastern region of Jiangsu Province of China, and it is a prime example of urbanization with its recent expansion of Suzhou Industrial Park (SIP), Suzhou New District (SND) and Taihu New Town. Established in 514 BC on the network of canals in the Yangtze floodplain, Suzhou has over 2500 years of history with its notable canals which attracts a large number of tourists due to its high cultural and rich historical significance [26,27]. Land use pattern and multiple data analyses were carried out to determine the relationships between physicochemical characteristics, culture-dependent microbiological parameters, an abundance of fecal markers and bacterial pathogens, land use patterns, and urbanization. As far as we are aware, this is the first detailed study to assess the impact of urbanization in this region using canals as a model system. These results will be useful for evaluating the effect of urbanization on the environment and public health, and for future urban development and pollution management. We hypothesized that urban intensification is positively related to eutrophication and microbial contaminations in urban surface water bodies, and this poses potential health risks to local residents.

## 2. Materials and Methods

### 2.1. Study Locations

**Figure 1:**
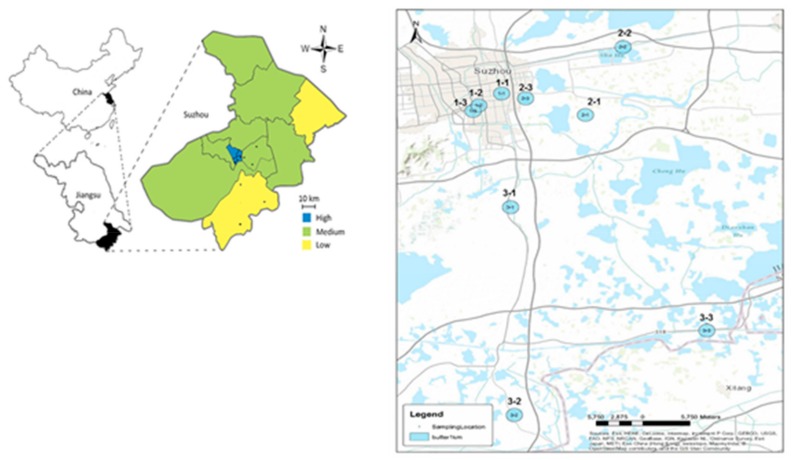
Sampling locations in Suzhou representing high, medium and low urban gradients (three each) selected for this study. Sampling locations are identified as 1-1, 1-2, 1-3 for high, 2-1, 2-2, 2-3 for medium and 3-1, 3-2 and 3-3 for low urban intensifications.

All sampling was conducted in or around the outskirts of Suzhou, and nine locations with three urban intensities (High, Medium and Low; Figure 1) were selected for the study. The urban intensity classification was based on population density/km^2^ for each category: >8000, 1700–2100 and 800–1100 persons/km^2^, respectively. Field sampling was carried out on four occasions in the winter and summer of 2016. In summer 2016, three sampling locations in the Huangshan area were selected as controls for this project. Huangshan is a natural reserve in a mountain region (approximately 500 km west of Suzhou), where population density, urban intensification and human activity are low compared to Suzhou, and the waterways in Huangshan area are protected by the local government. Both Huangshan and Suzhou have a subtropical monsoon climate (annual average temperature and rainfall ranges from 13 to 20 °C, and 800 to 1600 mm, respectively) with similar weather conditions further supporting their selection as appropriate control sites. Climatic data, including temperature and precipitation in Suzhou for 2015 and 2016 (Figure S1), were gathered from annual reports published by the local government [28,29] to explore the relationship with other water quality parameters observed in this study. The details of the sampling locations, including the geographic coordinates and land use patterns, are provided in Table S1.

**Figure 2:**
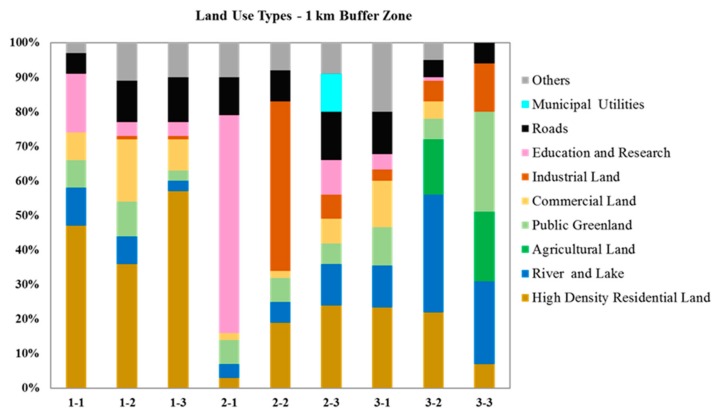
Land use types in high (1-1 to 1-3), medium (2-1 to 2-3) and low (3-1 to 3-3) urbanization locations. The land use type reported is for 1 km buffer zone.

Land use maps were prepared using ArcGIS 10.2 (Environmental Systems Research Institute, Inc. (ESRI), Redlands, CA, USA). On the basis of the Open Street Map of Suzhou city, two layers of buffer zones were created with radii of 500 m and 1000 m respectively around all sample points. By referencing the official land use maps of Gusu District, Suzhou Industrial Park District and Wujiang District of Suzhou as well as Google maps covering the sample areas, detailed land use types were digitized within these buffer zones in accordance with the national Code for classification of urban land use and planning standards of development land (GB50137-200), and the land use composition within the areas of buffer zones calculated (Figure 2). The specific explanation of each land use classification is provided in Table S2.

### 2.2. Field Sampling

Five liters of water were collected from each sampling location in sterile polypropylene containers. Parameters including water temperature and conductivity were measured in the field using a thermometer and an EC/TDS/TEMP WATERPROOF COMBO METER (COM-100) (HM Digital Inc. Culver City, CA, USA), respectively. The samples were transported to the laboratory for nutrients, microbiological and molecular analyses and processed within 8 h of sample collection. Samples which were used for nutrients and microbiological analyses were kept on ice until they were brought to the laboratory. Sampling was carried out at four time points in Suzhou, covering winter and summer to assess the impact of urbanization and seasonal variation on these parameters. In summer 2016, additional sampling was conducted in the Huangshan area to provide a control dataset. For each season, field sampling was conducted twice to ensure the veracity of water quality characteristics, and the samples were collected 6 weeks apart in a season. Water samples (500 mL for each) were filtered through 0.22 μm polycarbonate membrane filters (Millipore, UK) in triplicate to collect microorganisms for DNA extraction, and the filters were stored at −20 °C prior to extraction.

### 2.3. Physicochemical Analyses

Water temperature (WT), pH, conductivity (EC), total nitrogen (TN), total phosphorous (TP), nitrate nitrogen (NO_3_-N), nitrite nitrogen (NO_2_-N), ammonium nitrogen (NH_4_-N), phosphate (PO_4_-P), total organic carbon (TOC) and chlorophyll *a* were measured.

pH was measured using a Eutech pH 700 instrument (Thermo Fisher Scientific Inc., Waltham, MA, USA). TN and TP were measured by peroxodisulphate oxidation and spectrophotometric methods. NO_3_-N, NO_2_-N, NH_4_-N, and PO_4_-P were determined using a continuous flow analyzer (Skalar SA 1000, Breda, The Netherlands) [30]. TOC was measured with a Shimadzu analyzer (model 5000; Tokyo, Japan) by high-temperature oxidation. Chlorophyll *a* was measured as in suspended algae biomass in water samples based on the procedures of water and wastewater analysis used by the American Public Health Association [31].

### 2.4. Microbiological Analyses

#### 2.4.1. Culture-Dependent Methods

A total viable count (TVC) was carried out using plate count agar (PCA) [32]. Serial dilutions were made for collected water samples and 50 μL samples were plated on PCA plates in triplicate and incubated at 30 °C for 48 h. The colonies were counted to determine the average number of colony forming units (CFU) per mL.

Total coliforms (TC) in water samples were determined by the pour plate method [33]. For this, 100 or 200 μL samples of serial dilutions were plated on Harlequin^TM^ *E. coli*/coliform medium (LabM, Heywood, UK) plates in triplicate. Plates were incubated at 37 °C for 24 h and the number of *Escherichia coli* (*E. coli*) (blue-green colonies) and coliforms (rose-pink colonies) were counted to determine the average number of total colony forming units (CFU) per mL.

Thermotolerant coliform counts (TTC) in water samples were carried out by membrane filtration and pour plate methods. Serial dilutions were made and 2 mL samples filtered through 0.22 μm isopore^TM^ membrane filters (Merck Millipore Ltd. Tullagreen, Carrigtwohill Co. Cork, Ireland), which were then placed on mFC agar medium (containing 1% rosalic acid) (Difco, Sparks, MD, USA) plates in triplicate. The mFC (membrane fecal coliform) agar medium was formulated by Geldreich et al. to enumerate thermotolerant coliforms/fecal coliforms by membrane filter technique without prior enrichment [34]. Plates were incubated at 44.5 °C for 24 h and the colonies that exhibited blue shades were counted to determine the number of TTC colony forming units (cfu) per mL.

#### 2.4.2. Culture-Independent Methods (qPCR)

Genomic DNA was extracted from membrane filters using the PowerSoil DNA isolation kit (Mo Bio, Carlsbad, CA, USA) according to the manufacturer’s instructions and the filters were cut into pieces and placed into the PowerBead tubes aseptically. The extracted DNA was quantified using NanoDrop ND 2000C spectrophotometer (Thermo Scientific, Marietta, OH, USA), verified by gel electrophoresis and stored at −20 °C until further processing.

Quantitative polymerase chain reaction (qPCR) assays were performed to assess the total, human and avian associated fecal sources. All qPCR reactions were run in triplicate with a final reaction volume of 20 μL. The sequences of the primers and probes along with concentrations are presented in Table S3. The accuracy and efficiency of the standard curves were determined by including a positive control of 10^3^ copies of plasmid standard as unknown in each assay [35]. A seven-point 10-fold serial diluted plasmid DNA with the target sequence was used to generate a standard curve (with a range of 10^1^ to 10^7^ copies/reaction) in each qPCR assay.

Two TaqMan based assays (BacUni and HF183 Taqman) were selected for detection of total and human-associated *Bacteroidales*, and one SYBR-Green-based assay (GFD) was selected for detection of avian associated fecal markers [36,37,38]. These markers were validated previously for the Taihu watershed region by our research group [39]. Six qPCR assays targeting *Enterococcus*, *Arcobacter butzleri*, *Shigella* sp., *Campylobacter jejuni*, *Salmonella* spp. and Shiga toxin producing *E. coli* (STEC) were selected for this study and all these assays were based on TaqMan chemistry.

TaqMan based qPCR assays (20 μL of master mix) contained 10 μL of Premix Ex Taq^TM^ (Probe qPCR) (Takara Bio Inc.), 0.4 μL of ROX Reference Dye II (Takara Bio Inc.), 2μL of template DNA, 6 μL nuclease-free water and 2 μL of primers and probe set with the final concentrations as shown in Table S3. SYBR Green assays (20 μL of master mix) contained 10 μL of SYBR Green PCR master mix (Thermofisher Technologies, Foster City, CA, USA), 7.0 μL nuclease-free water, 2 μL of template DNA and 1 μL of primer mixture with a final concentration as shown in Table S3.

Plasmid DNA standards were constructed for all the qPCR assays targeting fecal markers and genes of pathogenic bacteria as described previously [39]. For quantification of fecal markers, the target genes were PCR-amplified from respective fecal DNA extracts (human and avian fecal samples) using the primers designed previously (Table S3). For pathogenic bacteria, the target genes were PCR-amplified from respective genomic DNA of target organisms (*Salmonella* ATCC 14028, *Arcobacter butzleri* ATCC 49616, *Campylobacter jejuni* sub sp. *jejuni* ATCC 29428, *Escherichia coli* ATCC 35150, *Shigella sonnei* ATCC 9290 and *Enterococcus* ATCC 29212) using the primers previously reported (Table S3). The purity and concentration of plasmid DNA were quantified using a NanoDrop ND 2000C UV spectrophotometer, and the gene copy number were calculated as described previously by Oster et al. [35].

In total, 36 water samples collected during four seasons (winter and summer 2015 and 2016) from nine sampling locations of Suzhou canals with three different urban gradients (and three water samples collected from Huangshan as control or pristine area during summer 2016) were investigated to assess the impact of urbanization on the detection frequency and abundance of fecal markers and bacterial pathogens.

### 2.5. Statistical Analyses

Two-way analyses of variance (ANOVA) were performed to study the variations of all physicochemical and microbiological parameters accordingly to seasons (winter and summer) and urban intensifications (high, medium and low) by using the software IBM SPSS Statistics 20 [40].

All the qPCR assays with R^2^ values of above 0.95 and efficiencies between 85 and 110% were considered as acceptable for detection and quantification of target markers in environmental samples. The details of the amplification efficiency, the linear range of quantification (R^2^), the limit of detection (LOD), the limit of quantification (LOQ) and final assessment of qPCR results for each fecal marker and pathogen assays are provided in Table S4. The qPCR results for each assay of fecal markers and pathogens were processed based on LOD in Table S4 as described in previous studies [35,41]. For statistical analysis, the abundance of MST markers and genes of pathogenic bacteria were log transformed and non-detects (NDs) were substituted with 1/2 limit of detection (LOD) as described previously [42].

## 3. Results

### 3.1. Variation in Physicochemical Parameters

Water temperature (WT) was higher in summer than in winter (Table 1). The WTs were higher in locations with medium urbanization as compared to high and low. The pH values of the sampling locations ranged from 7–8 (with few exceptions—pH values were 8.71 and 8.16 for samples collected at 3-3 and H-2 collected in summer 2016, respectively) (Table 1). The pH values recorded during field sampling showed significantly lower level among the sampling locations with high (7.1–7.9) urban intensification than medium (7.4–7.9) and low (7.3–8) urban intensification, however, no significant differences between seasons were observed (Table S5). Conductivity values were significantly higher in winter than in summer. The conductivity values were significantly higher in high (393–832 μS/cm) and medium (389–615 μS/cm) than low (186–573 μS/cm) urbanization locations and these values were extremely high as compared to values recorded in samples collected from the natural reserve mountain in Huangshan (45.6–146 µS/cm) (Table 1).

**Figure 3:**
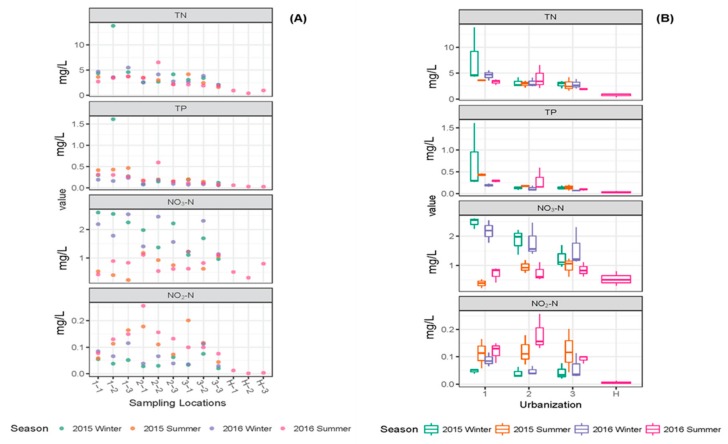
The variation in total nitrogen (TN), total phosphorus (TP), nitrate-N (NO_3_-N) and nitrite-N (NO_2_-N) values observed in different sampling locations and seasons. The individual values for each parameter (**A**) and boxplots (**B**) with median value (line within each box), quartile interval (box), the minimum and maximum value (whiskers) are shown.

**Figure 4:**
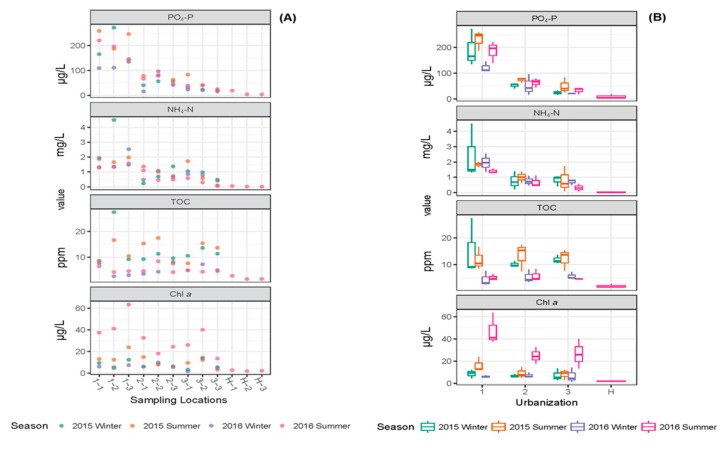
The variation in phosphate (PO_4_-P), ammonia-N (NH_4_-N), total organic carbon (TOC) and chlorophyll *a* (Chl *a*) values observed in different sampling locations and seasons. The individual values for each parameters (**A**) and boxplots (**B**) with median (line within each box), quartile interval (box), minimum and maximum value (whiskers) are shown.

Changes in the nutrients such as TN, TP, NO_3_-N, NO_2_-N, PO_4_-P and NH_4_-N at different locations and during different seasons are shown in Figure 3 and Figure 4.

The key water quality parameters TN, TP, NH_4_-N, PO_4_-P and TOC were extremely high in the second location (1-2) in high urbanization region during winter 2015 (Table 1, Figure 3 and Figure 4). The statistical analysis (Two-way ANOVA) showed that significant variation between locations (urbanization) was observed for the parameters TN, TP, NH_4_-N and PO_4_-P (Table S5); among the three urban intensifications (high, medium and low) in Suzhou, the variation in the nutrient values were observed mainly between high vs. medium and high vs. low, and no significant variations in the parameters (except for PO_4_-P) were observed between medium vs. low (Table S6). In addition to the variation between the sampling locations, TN and PO_4_-P values showed significant variation between seasons as well. No interaction between season and urbanization was observed based on two-way ANOVA (Table S5). However, the parameters NO_3_-N, NO_2_-N and chlorophyll *a* showed significant variation with respect to season and not the degree of urbanization (Table S5). Chlorophyll *a*, as an indicator of algal growth in water bodies, was extremely high in location 2-2 in summer 2016 (750 µg/L, omitted from Table and Figure to avoid the influence of this value to the whole dataset). The parameter TOC did not show significant variation either between sampling locations or across seasons (Table S5). As shown in Table 1 and Figure 3 and Figure 4, the nutrient values observed in samples from Huangshan were extremely low as compared to Suzhou canals, indicating good water quality in the absence of any influence from urbanization and other anthropogenic activities.

### 3.2. Variation in Microbiological Parameters

#### 3.2.1. Culture-Dependent Microbiological Parameters

**Figure 5:**
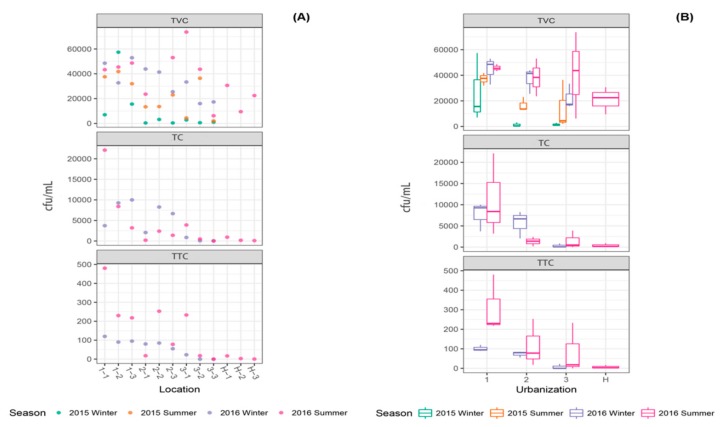
The variation in the total viable count (TVC), total coliform (TC) and thermotolerant coliform (TTC) values observed in different sampling locations and seasons. The individual values for each parameters (**A**) and boxplots (**B**) with median value (line within each box), quartile interval (box), minimum and maximum value (whiskers) are shown.

As one of the culture-dependent microbiological parameters, TVC was measured to quantitatively assess the microbial load in water samples collected from sampling locations in Suzhou and Huangshan (Figure 5). TVC showed significant variation with respect to urbanization (Table S5), and high TVC values were observed in locations with high (7–57.4 × 10^3^ cfu/mL) urbanization, as compared to locations with low (0.6–73.7 × 10^3^ cfu/mL) urbanization and Huangshan (9.5–30.7 × 10^3^ cfu/mL) (Table S6).

TC showed significant variation with respect to sampling locations in Suzhou (Table S5) and the TC numbers were higher (3.2–22.1 × 10^3^ cfu/mL) in locations in highly urbanized regions compared to low urbanized regions (0–3.9 × 10^3^ cfu/mL) (Table S6). Extremely low levels (0.1–0.9 × 10^3^ cfu/mL) of TC counts were observed in water samples collected from Huangshan (Table 1, Figure 5).

The TTC count showed significant variation with respect to both urbanization and season (Table S5). Higher TTC counts (90–480 × 10^3^ cfu/mL) were observed in locations with high urbanization as compared to locations with low (0–233 × 10^3^ cfu/mL) urbanization (Table S6). In water samples collected from Huangshan, extremely low TTC counts (0.5–17 × 10^3^ cfu/mL) were observed (Table 1, Figure 5). The TTC count results mirrored the TC counts well, and either TC or TTC may be used to assess the microbiological quality of canal water. Both median and 95th percentile values of the microbiological parameters particularly TC and TTC measured at different urban gradients support the above results; TC and TTC had high median and 95th percentile values in the water samples collected from high urbanization locations as compared to medium and low urbanization locations in Suzhou and Huangshan (Table S7).

#### 3.2.2. Detection and Quantification of Fecal Markers

**Figure 6:**
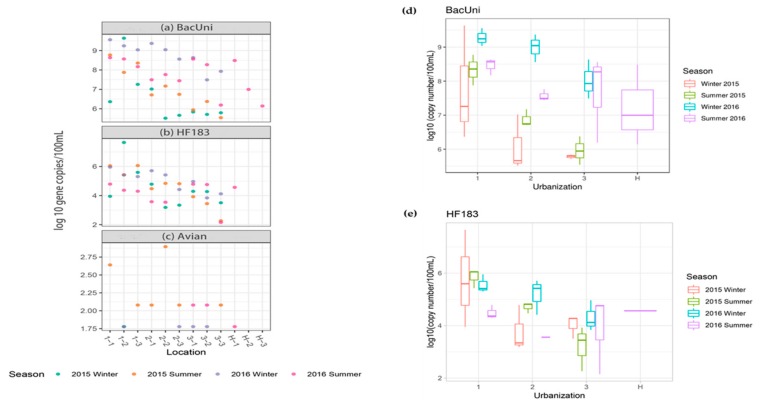
Concentration of total *Bacteroidales* (**a**), human-associated *Bacteroidales* (**b**), and avian associated markers (**c**) in water samples collected from different locations in Suzhou and Huangshan. Comparison of fecal marker concentrations in Suzhou canals across different urban intensifications and Huangshan. (**d**) Total *Bacteroidales* and (**e**) human-associated *Bacteroidales*. The left panel shows individual values for each parameters (a–c) and the right panel shows boxplots (d and e) with median value (line within each box), quartile interval (box), minimum and maximum value (whiskers).

The total *Bacteroidales* marker was detected in all the water samples (100%), and in general, higher levels were observed in water samples collected from locations with high (6.37 to 9.63-log_10_ gene copies/100 mL) urbanization than medium (5.52 to 9.37-log_10_ gene copies/100 mL) and low (5.54 to 8.63-log_10_ gene copies/100 mL) urbanization (Figure 6a). A significantly higher level of total *Bacteroidales* marker was observed among the locations with high urbanization (*p* = 0.008) than medium and low (Figure 6d, Tables S5 and S6). For water samples collected from Huangshan, low levels of total *Bacteroidales* were observed (6.14 to 7.00-log_10_ gene copies/100 mL) except at location H-1, where the numbers were 8.49-log_10_ gene copies/100 mL.

Human-associated *Bacteroidales* markers were frequently detected in most of the samples tested (36 out of 39 water samples, 92%), and the concentrations ranged from 2.15 to 7.65-log_10_ gene copies/100 mL (Figure 6b). Similar to total *Bacteroidales*, significantly (*p* = 0.002) higher levels of human-associated *Bacteroidales* were observed in water samples collected from locations with high urbanization (3.95 to 7.65-log_10_ gene copies/100 mL) than medium (3.19 to 5.71-log_10_ gene copies/100 mL) and low (2.15 to 4.97-log_10_ gene copies/100 mL) urbanization (Figure 6e, Tables S5 and S6). For water samples collected from Huangshan, human-associated *Bacteroidales* was only detected in location H-1 (4.57-log_10_ gene copies/100 mL) but not at H-2 and H-3 locations. The results of the abundance of human-associated *Bacteroidales* showed the same pattern as the total *Bacteroidales* marker numbers, the only obvious difference is that the concentrations of total *Bacteroidales* were usually 10^2^–10^3^ fold higher than the concentrations of human-associated *Bacteroidales*.

Avian fecal marker (GFD) was only detected in 14 out of 39 water samples (36%), however, they were at the quantifiable range in two water samples only (2.64 to 2.89-log_10_ gene copies/100 mL) (Figure 6c).

#### 3.2.3. Frequency of Detection and Abundance of Genes of Bacterial Pathogens

**Figure 7:**
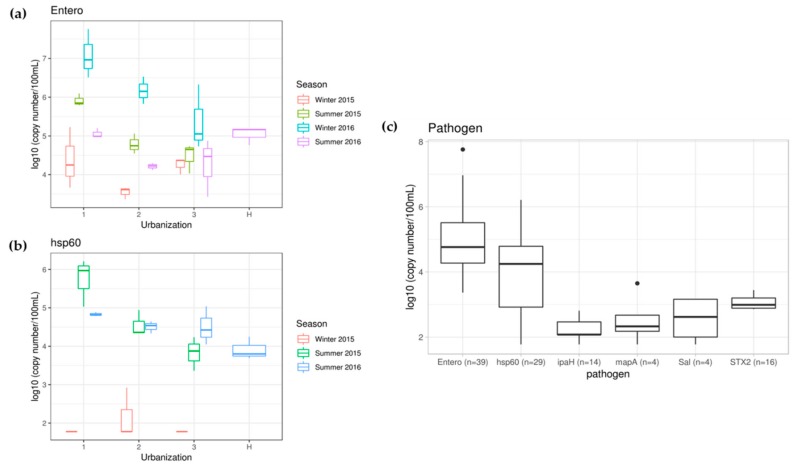
The abundance of *Enterococcus* spp. (**a**), *Arcobacter butzleri* (**b**) and comparison of six pathogens (**c**) in water samples collected from Suzhou canals and streams in Huangshan.

The most frequently detected genes of bacterial pathogens were *ENT1A* (100%; *Enterococcus*), followed by *hsp60* (74%; *Arcobacter butzleri*), *STX2* (41%; STEC), *ipaH* (36%; *Shigella* sp.) *mapA*, (10%; *Campylobacter jejuni*) and *Salmonella* spp. (10%) (Table 2). Considering the limit of quantification (LOQ) as the selection criteria, a gene specific to *Enterococcus* was detected in all the water samples (100%), and the concentrations ranged from 3.37 to 7.76-log_10_ gene copies/100 mL (Table S8A and Figure 7a). Significantly (*p* = 0.034) higher levels of *Enterococcus* specific genes were observed in water samples collected from locations with high urbanization (Table S5). The *Enterococcus* qPCR data followed the trend of TC, TTC, total and human-associated *Bacteroidales* data. The qPCR *targeted to Arcobacter butzleri* showed significantly higher (*p* = 0.000) levels of the *hsp60* gene in summer than in winter, and also significantly (*p* = 0.005) higher levels were observed in water samples collected from locations with high urbanization than low urbanization. All the water samples collected in summer were quantified (3.36 to 6.21-log_10_ gene copies/100 mL), but only one sample (2-3) was quantified (2.92-log_10_ gene copies/100 mL) in winter 2015 (though seven more samples were detected but not quantifiable) and none of the samples collected from winter 2016 were at detectable levels (Table S8B and Figure 7b). The highest concentration (6.21-log_10_ gene copies/100 mL) was observed at location 1-3 in summer 2015. Although the genes of other pathogens (*Shigella* sp., *Campylobacter jejuni*, *Salmonella* spp. and Shiga toxin producing *E. coli*) were detected in few samples (10%–41%), they were quantifiable only in fewer samples (2.31 to 3.65-log_10_ gene copies/100 mL) and most of these samples were collected from high urbanization locations (Table S8C–F, Figure 7c). In general, a high level of pathogens was observed mainly in locations with high urbanization than medium and low (Table S6).

## 4. Discussion

Urbanization can cause major changes to freshwater systems, such as increasing chemical and microbial contaminations and eutrophication [43]. In the present study, the impact of urban intensification on the physicochemical and microbiological characteristics of surface water was studied using canals as model systems. Earlier studies have shown that artificial water systems such as canals are sensitive to anthropogenic inputs from human and industrial activities [44]. Therefore, canals represent a strong model system to study the impact of urbanization on general water quality and the data presented here provide further support for this position.

Since the field sampling was carried out at different time points on the same day (morning, noon or afternoon), the variation of the water temperature with respect to sampling locations in Suzhou observed could be due to the sampling time. The pH values varied significantly between sampling locations but not with seasons. pH is important for aquatic life as it determines the solubility and bioavailability of chemicals including nutrients and heavy metals [45], and most of the pH values were within an acceptable range (6.5–8.5) as determined by the Chinese Ministry of Environment Protection (MEP) [46]. The variation in the pH between sampling locations could be due to differences in the nutrient levels and input from the surroundings (land use) but the values obtained here are consistent with the results reported by Yu et al. (2012) for surface water quality of the Grand Canals. EC values were extremely high in high urbanization locations, most likely reflecting the amount of dissolved salts, total dissolved solids and inorganic compounds present in the water samples [47]. EC is one of the important parameters to assess the water quality and it is an indirect indicator of water pollution particularly wastewater or sewage discharge [48]. The presence of wastewater or domestic sewage can raise the EC in surface water due to the presence of phosphate, nitrate, chloride and other ions [48,49], which is another likely driver of high conductivity observed in urbanized locations.

The surface water quality in China is classified into six grades [46]: Grade I-III are applicable to the water from sources or protected areas for centralized sources for drinking and such grades could be considered as good quality; Grade IV and V are applicable to water bodies for industrial and agricultural use, and such grades could be considered as moderately polluted; Grade V+ means seriously polluted. TN concentrations were high in almost every location in Suzhou (highest in high and medium urbanized locations) as compared to the MEP standards (Grade V+: TN > 2 mg/L), which indicates that these locations were seriously polluted with multiple sources. TN concentrations were low and within the limit in control locations in Huangshan (0.29–1.17 mg/L). TP and ammonium concentrations were beyond the standards (Grade V+: TP > 0.4 mg/L, ammonium nitrogen > 2 mg/L) especially in all the locations with high urbanization (0.13–2.10 mg/L of TP and 1.01–7.84 of NH_4_-N). Nutrients such as TN, TP, NH_4_-N and PO_4_-P varied significantly with sampling locations and, with the exception of TP, across seasons (Table 1). Elevated levels of nutrients were consistently observed in high urbanization areas. Although some of the nutrients come from natural processes, wastewater discharge, leakage of domestic sewage, agricultural runoff (fertilizers) and industrial wastes typically cause nutrient increases in water bodies, leading to eutrophication and excessive algal growth in lakes and reservoirs [50]. High concentrations of multiple nutrients observed in high urbanization locations were correlated with the land use types. The dominant land use type in the high urbanization locations was high-density residential land (36%–57%) followed by commercial land (8%–18%). The highest nutrient enrichment on most occasions was found at location 1-2, which is a closed canal system with reduced water flow and is bordered by a high-density residential area. In medium urbanized locations (2-1 to 2-3), the dominant land use types were research and education institutions as well as associated residential areas (0%–63%), industrial land (0%–49%) and high-density residential land (3%–24%). In contrast, the land use types in low urbanization locations were rivers and lakes (11%–34%), agricultural land (0%–20%), public green land (6%–29%) and high-density residential land (7%–21%) followed by industrial land (3%–14%). The main source of nutrients observed in low urbanized locations (3-1 to 3-3) could be agricultural runoff followed by domestic wastewater and industrial wastes. A high number of transportation activities by ferries were also a feature at two of the low urbanization locations (3-1 and 3-3) which could have increased water turbidity by resuspension and thus incorporation of ionic sediment material.

The surface water quality of the Grand Canal has been found to harbor high concentrations of nitrogen, phosphorus and various metals [20]. It was also reported that water quality deterioration in the water system of Shanghai was primarily due to the presence of nutrients such as ammonia nitrogen, and low dissolved oxygen levels [51]. With continued urbanization, wastewater and pollutants from household consumption have had a major influence on emission loads in Shanghai [52]. Meanwhile, altered land use and land cover (LULC) generally has been associated with impacts on the flow and water quality at multiple spatial scales [53,54]. The relationship between land use and lake/river water quality or impact on urban water quality has been the subject of other studies in China [51,55], and generally reflects the results found in the present study with decreasing water quality with increasing urbanization.

Overall, Chl *a* concentrations did not vary between sampling locations but were higher in summer correlating with increased temperature. The Chl *a* concentration was extremely high in location 2-2 in summer 2016, which is correlated with high algal growth observed in this location during sample collection. High algal growth is caused by eutrophication of water bodies and the extremely high concentrations of TN and TP (6.54 mg/L, 0.59 mg/L, respectively) recorded at location 2-2 in summer 2016 indicated serious eutrophication.

Since high-density residential land was the dominant land use pattern in locations with high urbanization, higher population density coupled with human activities and domestic wastewater/sewage undoubtedly contributed to high TVC, TC and TTC values in high urbanization locations. All these culture-dependent microbiological assays (TVC, TC and TTC) essentially showed similar results in this study. Total coliform (TC) numbers were measured to assess the sanitary quality of the water. As the coliform group of bacteria largely originate from the digestive tracts of warm-blooded animals, their presence in water samples indicates bacteria of fecal origin although coliform themselves do not typically cause serious illnesses or diseases [47]. Thermotolerant coliform (TTC) or fecal coliform is a group of anaerobic facultative bacteria whose presence in high numbers indicates fecal contamination and increased health risk [56], however, their presence is not always an absolute indicator of fecal contamination or the presence of harmful bacteria in water samples [57] (Figure 5). Glinska-Lewczuk et al. [58] observed an increase in nutrient concentration and indicator bacteria (heterotrophic plate count and thermotolerant coliforms) associated with urbanized sections of a lowland river in Poland. Santiaogo-Rodriguez et al. (2016) reported that fecal indicator bacteria positively correlated with urbanization and rainfall events in a tropical watershed in Puerto Rico [59].

Fecal pollution of surface waters is a serious issue for aquatic ecosystems and human health. Fecal material enters into the freshwater ecosystem from several sources such as effluents from wastewater treatment plants, septic leaks, urban and storm runoff water [60]. Total *Bacteroidales* and human-associated *Bacteroidales* increased with increasing urbanization. Total *Bacteroidales* (BacUni) were obviously richer (10^2^–10^3^ fold) than human-associated *Bacteroidales* (HF183) in water samples, although avian associated markers did not show any trend, and were found in low densities. These findings indicate that canals in high urbanization areas are affected by fecal pollution and human sewage. This could be due to the discharge of sewer and septic waste in the water bodies directly or indirectly [61]. Runoff after rainfall could also lead to fecal source entry into watersheds [62]. However, the higher precipitation in summer than in winter in Suzhou was not associated with either BacUni or HF183, which indicated that their presence and abundance were not affected by rainfall. Similar trends of water impairment due to urbanization have been reported elsewhere for an urbanized tropical watershed [63]. It was also confirmed by Molina et al. (2014) that high levels of human contamination were detected in urban runoff [64]. Since almost no poultry or other related animal activities were observed near the sampling locations, the avian host-associated marker was only quantifiable in some samples and at very low concentrations. Huangshan represented a good control area as the waterways are protected by the local government and the area has considerably lower human population density. Low levels of fecal markers were observed in water samples collected from there, except in location H-1 which is close to a village at the foot of the mountain where some small-scale farming activities (e.g., poultry) were observed during sampling, which caused some fecal contamination at location H-1.

Higher levels of *Enterococcus* spp. were observed in water samples collected from locations with high urbanization in Suzhou. Many species of *Enterococcus* are prevalent in the gastrointestinal tract of mammals, making them widely used as bacterial indicators for fecal pollution in water. Although *Enterococcus* spp. usually do not pose any health risks to humans, their presence in water could suggest the possible presence of enteric pathogens [65]. Therefore, *Enterococcus* spp. values were related to the results of fecal markers, especially human-specific fecal markers, and exhibited similar distributions to TC and TTC. The optimal growth temperature range for *Arcobacter butzleri* is from 26 to 30 °C [66], which matches with the summer temperature observed in Suzhou canals (27–33 °C) (Table 2 and Figure S1) therefore this bacterium was detected in the samples collected during summer but was only detectable in few samples collected in winter. The genus *Arcobacter* was reported to be associated with human illness and fecal contamination, and human fecal sources were likely to be a key contributor to *Arcobacter* contamination [67]. Some species of this genus are considered as emerging food pathogens [68] and among these species, *A. butzleri* was an underestimated enteropathogen [69]. *Shigella* sp. is one of the major food-borne pathogens that caused human shigellosis worldwide [70]; Shiga toxin producing *E. coli* (STEC) are associated with the production of Shiga toxin, and STEC play an important role as pathogens in humans [71]; *Campylobacter jejuni* is a clinically important bacterial species [72]; *Salmonella* spp., a leading cause of morbidity and mortality due to food and waterborne diseases in many countries, causes gastroenteritis, typhoid and diarrheal illnesses for human beings [70]. However, all these four pathogens were only quantified in several samples at very low levels as compared to *Enterococcus* spp. and *Arcobacter butzleri* and these pathogens were detected in high levels in locations with high urbanization in Suzhou.

## 5. Conclusions

In this study, significantly higher levels of multiple nutrient parameters (TN, TP, NH_4_-N and PO_4_-P), microbial load (TVC, TC and TTC), fecal markers (total *Bacteroidales* and human-associated *Bacteroidales*) and bacterial pathogens (*Enterococcus* spp. and *Arcobacter butzleri*) were observed in water samples collected from locations with high urban intensification as compared to medium and low urbanization in Suzhou canals and control locations of Huangshan. Land use types of locations with high urban intensification were mainly high-density residential lands. Therefore, domestic wastewater or sewage was the main pollutant entering into city canals, causing serious eutrophication and microbial contaminations in those locations. The results obtained in this study conclude that urbanization impacts the water quality with high levels of nutrients and microbial load including fecal markers and pathogens in Suzhou canals, which have significance for public health. Microbial community analysis by next-generation sequencing (NGS) will be carried out for water samples to collect comprehensive microbiological data.

## Figures and Tables

**Table 1:** Physicochemical and microbiological characteristics of water samples collected from nine sampling locations across three urban intensifications in winter and summer 2015 and 2016. Samples from the control locations (Huangshan) were collected in Summer 2016. The results of the statistical analysis (two-way analysis of variance (ANOVA)) is also shown in this table.

Parameters	Winter 2015 and 2016 Range (Min-Max)			Summer 2015 and 2016 Range (Min-Max)			Control Location (Huangshan)	*p* Values	
	High	Medium	Low	High	Medium	Low		Urbanization	Season
Water temp. °C	6–11	5.1–9.8	5.9–10	28–34.1	26–33.4	28.8–34.4	24.3–28.2	0.001 **	<0.001 ***
pH	7.1–7.9	7.51–7.9	7.58–7.9	7.3–7.7	7.39–7.86	7.3–8.71	7.12–8.16	0.006 **	0.178
Conductivity (µS/cm)	422–832	474–615	395–573	393–534	389–544	186–563	45.6–146	0.013 *	<0.001 ***
TN (mg/L)	2.85–16.5	2.14–4.48	1.57–4.21	2.11–4.67	1.92–10.58	0.96–4.63	0.29–1.17	0.001 **	0.038 *
TP (mg/L)	0.13–2.10	0.07–0.21	0.04–0.26	0.23–0.53	0.12–1.04	0.06–0.21	0.02–0.06	<0.001	0.102
NO_3_-N (mg/L)	1.01–3.42	1.13–2.56	0.83–2.83	0.17–1.01	0.05–1.25	0.24–1.96	0.21–0.92	0.816	<0.001 ***
NO_2_-N (mg/L)	0.01–0.14	0.02–0.08	0.01–0.12	0.05–0.20	0.06–0.35	0.03–0.27	0.00–0.02	0.422	<0.001 ***
PO_4_-P (μg/L)	48–497	14.37–121.51	11.71–46.45	92.78–315	30.44–117	16.11–88.9	3.44–28.93	<0.001 ***	0.002 **
NH_4_-N (mg/L)	1.01–7.84	0.18–1.44	0.41–1.47	0.52–2.40	0.23–1.48	0.03–2.05	0.01–0.10	<0.001 ***	0.148
TOC (mg/L)	1.99–42.3	3.17–13.23	3.55–13.8	3.72–20.8	3.75–23	3.67–15.6	1.31–3.35	0.936	0.745
Chlorophyll *a* (µg/L)	2.33–21.4	1.37–15.41	1.47-–6.56	3.39–68.86	2.7–50.177	1.95–54.42	0.95–3.17	0.329	<0.001 ***
Total viable count (× 10^3^ cfu/mL)	7–57.4	0.4–43.9	0.6–33.4	32–48.7	13.4–53.1	2–73.7	9.5–30.7	0.040 *	0.055
Total coliform count (× 10^3^ cfu/mL)	3.733–10	2.067–8.267	0.067–0.867	3.2–22.1	0.2–2.4	0–3.9	0.098–0.933	0.006 **	0.696
Thermotolerant coliform count (cfu/mL)	90–120	55–85	0–23	218–480	18–253	0–233	0.5–17	0.036 *	0.032 *

* Statistically significant difference at *p* < 0.05; ** Statistically significant difference at *p* < 0.01; *** Statistically significant difference at *p* < 0.001.

**Table 2:** Detection frequencies of pathogenic bacterial genes in water samples collected from Suzhou canals and streams in Huangshan.

Sample Type	No. of Samples Tested (*n*)	No. of Positive Samples ^a^					
		*Enterococcus* spp.	*Arcobacter butzleri* (*hsp60*)	*Shigella* (*ipaH*)	*Campylobacter* (*mapA*)	*Salmonella spp.*	*STEC* (*stx2*)
**Suzhou**							
Winter 2015	9	9 (100%)	8 (89%)	2 (22%)	1 (11%)	0	1 (11%)
Summer 2015	9	9 (100%)	9 (100%)	3 (33%)	0	1 (11%)	0
Winter 2016	9	9 (100%)	0	2 (22%)	3 (33%)	2 (22%)	7 (78%)
Summer 2016	9	9 (100%)	9 (100%)	6 (67%)	0	1 (11%)	6 (67%)
**Huangshan**							
Summer 2016	3	3 (100%)	3 (100%)	1 (33%)	0	0	2 (67%)
**Total**	39	39 (100%)	29 (74%)	14 (36%)	4 (10%)	4 (10%)	16 (41%)

^a^ Considering DNQ’s (detected, not quantifiable) as positive samples.

## Supplementary Materials

The following are available online at https://www.mdpi.com/1660-4601/16/10/1739/s1, Figure S1: The average air temperature and precipitation in Suzhou for each month during 2015 (A) and 2016 (B), Table S1: Description of sampling locations in Suzhou and Huangshan along with geographic coordinates and corresponding land use types, Table S2: The specific explanation of each land use classification in land use type analysis, Table S3: qPCR primers and probes used in this study for quantification of fecal markers and bacterial pathogens. Table S4: The limit of detection (LOD), limit of quantification (LOQ) and final assessment of qPCR results for each fecal marker and pathogen assays, Table S5: Two-way ANOVA of physicochemical and microbiological parameters and abundance of fecal markers and pathogenic bacteria for water samples, Table S6: Urbanization variation of physicochemical and microbiological parameters and abundance of fecal markers and pathogenic bacteria for water samples, Table S7: The median and 95th percentile data of TVC, TC and TTC for each urbanization gradient (High, Medium, Low) in Suzhou and Huangshan, Table S8A–F: Concentration of pathogenic bacterial genes (*Enterococcus* spp., *Arcobacter butzleri, Shigella, Campylobacter jejuni,* *Salmonella* spp. and STEC) in water samples collected from Suzhou canals and streams in Huangshan.
