# Supplementary material for: Urbanization Impacts the Physicochemical Characteristics and Abundance of Fecal Markers and Bacterial Pathogens in Surface Water
Source: Int J Environ Res Public Health. 2019 May 16;16(10):1739. doi: 10.3390/ijerph16101739 (PMC6572354; doi:10.3390/ijerph16101739)
Supplement: Supplementary file 1 [file ijerph-16-01739-s001.pdf]

Supplementary Materials

Urbanization Impacts the Physicochemical Characteristics and Abundance of Fecal Markers and Bacterial Pathogens in Surface Water

Tianma Yuan[1], Kiran Kumar Vadde[1], Jonathan D. Tonkin[2], Jianjun Wang[3], Jing Lu[4], Zimeng Zhang[5], Yixin Zhang[6], Alan J. McCarthy[7] and Raju Sekar[1,*]

[1] Department of Biological Sciences, Xi'an Jiaotong-Liverpool University, Suzhou 215123, China; Tianma.Yuan@xjtlu.edu.cn (T.Y.), Kumar.Kiran@xjtlu.edu.cn (K.K.V)

[2] School of Biological Sciences, University of Canterbury, Christchurch 8140, New Zealand; jdtonkin@gmail.com

[3] Nanjing Institute of Geography and Limnology, Chinese Academy of Sciences, Nanjing 210008, China; jjwang@niglas.ac.cn

[4] Futurepolis LLC, Suzhou 215021, China; jinglu.lyu@gmail.com

[5] Institute of Integrative Biology, University of Liverpool, Liverpool L69 3BX, UK; zimeng.zhang@liverpool.ac.uk

[6] Department of Health and Environmental Sciences, Xi'an Jiaotong - Liverpool University, Suzhou 215123, China; Yixin.Zhang@xjtlu.edu.cn

[7] Microbiology Research Group, Institute of Integrative Biology, University of Liverpool, Liverpool L69 7ZB, UK; aj55m@liverpool.ac.uk

* Correspondence: Sekar.Raju@xjtlu.edu.cn; Tel.: +86-512-8816-1656

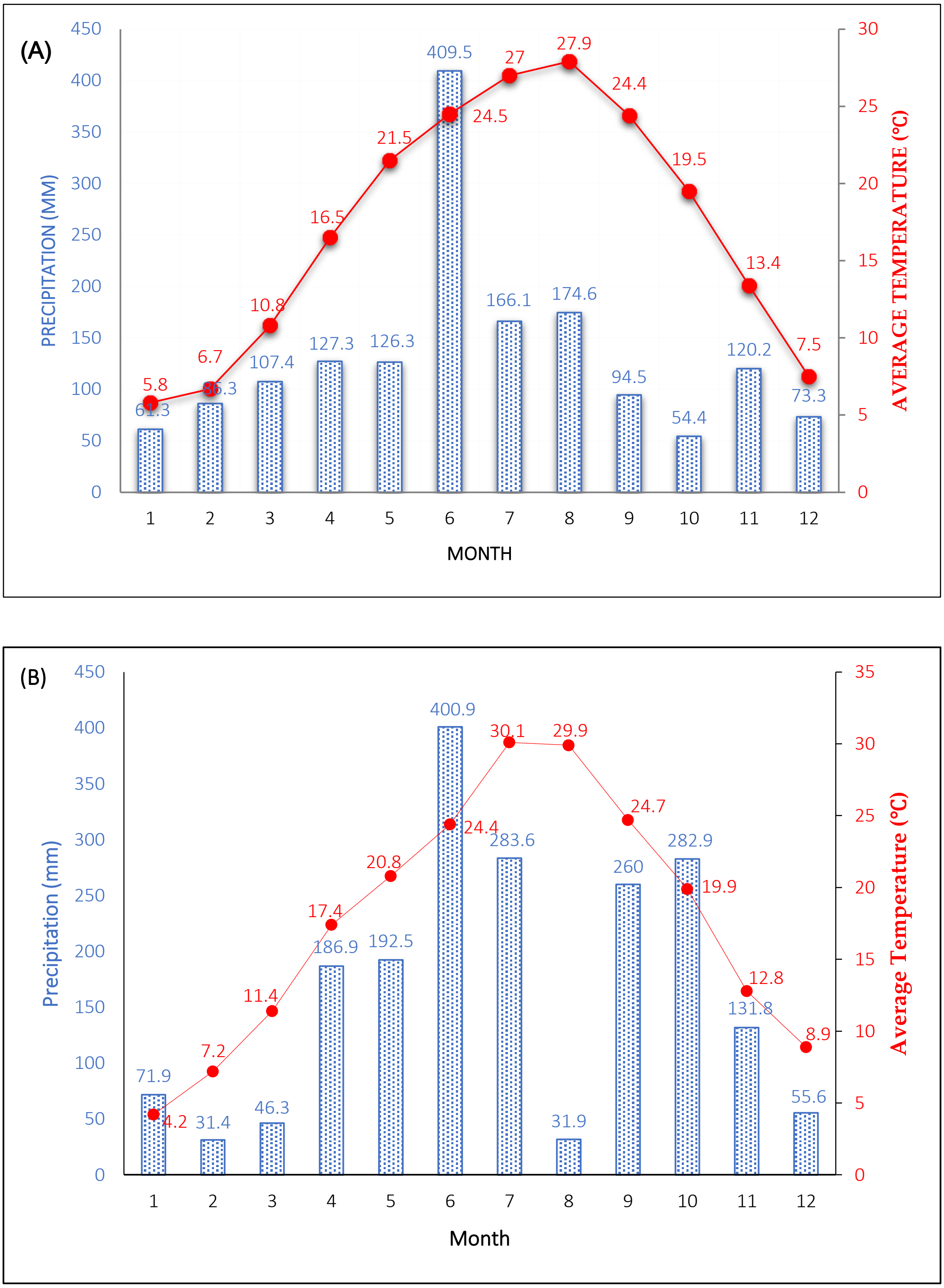

Figure S1. The average air temperature and precipitation in Suzhou for each month during 2015 (A) and 2016 (B).

Table S1. Description of sampling locations in Suzhou and Huangshan along with geographic coordinates and corresponding land use types*.

Sampling location	Description of location and land use types	Coordinates	
		Latitude	Longitude
1-1	Suzhou old town; High Density Residential Land (47%), Education & Research (17%), River and lake (11%), Commercial Land (8%), Public Greenland (8%), Road (6%), Reserved Land (3%)	N31°17′49″	E120°38′42″
1-2	Suzhou old town; High Density Residential Land (36%), Commercial Land (18%), Road (12%), Public Greenland (10%), River and Lake (8%), Education Research (4%); Others (12%).	N31°16′58″	E120°37′13″
1-3	Suzhou old town; High Density Residential Land (57%), Road (13%), Commercial Land (9%), Education and Research (4%), River and Lake (3%), Public Greenland (3%) and Others (11%).	N31°16′32″	E120°36′32″
2-1	Suzhou Industrial Park; Education & Research (63%), Road (11%), Reserved Land (9%), Public Greenland (7%), River and Lake (4%), High Density Residential Land (3%) and Others (3%)	N31°16′19″	E120°44′17″
2-2	Suzhou Industrial Park; Industrial Land (49%), High Density Residential Land (19%), Road (9%), Public Greenland (7%), Protective Greenbelt (7%) and Others (9%).	N31°21′9″	E120°46′51″
2-3	Suzhou Industrial Park; High Density Residential Land (24%), Road (14%), River and Lake (12%), Municipal Utilities (11%), Education and Research (10%), Industrial Land (7%), Commercial Land (7%), Low Density Residential Land (7%), Public Greenland (6%) and Others (2%)	N31°17′26″	E120°40′21″
3-1	Suzhou outskirt; High Density Residential Land (21%), Commercial Land (12%), River and Lake (11%), Reserved Land (11%), Road (11%), Public Greenland (10%), Education and Research (4%), Residential Land (3%), Industrial Land (3%), Hospital (3%) and Others (11%)	N31°09′46″	E120°39′18″
3-2	Suzhou outskirt; High Density Residential Land (22%), River and Lake (34%), Agriculture land (16%), Public Greenland (6%), Commercial Land (5%), Low Density Residential Land (5%), Road (5%) and Others (7%)	N30°54′53″	E120°39′40″
3-3	Suzhou outskirt; Public Greenland (29%), River and Lake (24%), Agricultural Land (20%), Industrial Land (14%), High Density Residential Land (7%), Road (6%).	N31°0′55″	E120°52′19″
H-1	Close to village at the foot of the mountain; Farmland, Family	N30°16′13″	E118°4′45″

	farming activities (e.g. poultry)		
H-2	Close to the roads to the mountain; Protected nature area.	N30°10′15″	E118°3′32″
H-3	In the mount mountain; Protected nature area.	N30°6′28″	E118°1′40″

* Land use in 1 km buffer zone.

Table S2. The specific explanation of each land use classification in land use type analysis.

No.	Land Use Classification	Explanation
**1**	Administrative Land	Government agencies, non-profit organizations and other facility land
**2**	Commercial and Residential Mixed Land	Land used for both commercial and residential land
**3**	Commercial Land	All sorts of commercial, business and entertainment use facility
**4**	Cultural Entertainment Land	Libraries, exposition etc. cultural facility land
**5**	Education & Research Land	Higher-education institution, secondary technical education institution, middle schools, primary schools, research institutions, as well as affiliated dormitories
**6**	High Density Residential Land	Middle to High rise residence with relatively complete amenity
**7**	Hospital	Hospital, health care, habitation related land
**8**	Industrial Land	Industry and mining factories
**9**	Low Density Residential Land	Low-rise residence
**10**	Protective Greenbelt	Greenland functions as sanitation and safety buffers
**11**	Municipal Utilities	Land for facilities providing services, environment and safety
**12**	Public Greenland	Open for public primarily for recreational purpose
**13**	Reserved Land	Land reserved for future use
**14**	River & Lake	Non-development land, all sorts of water bodies
**15**	Road	Urban road and traffic facility land
**16**	Sports	Sports facility and training facility land

Table S3. qPCR primers and probes used in this study for quantification of fecal markers and bacterial pathogens.

Target source/organism	Assay	Primer/Probe	Sequence (5′-3′)	Concentration (nM)	Product size (bp)	Annealing temp (°C)	References
Total *Bacteroidales*	BacUni	BacUni-520F	CGTTATCCGGATTTATTGGGTTTA	400	170	60	[1]
		BacUni-690R	CAATCGGAGTTCTTCGTGATATCTA	400			
		BacUni-656P	FAM-TGGTGTAGCGGTGAAA-MGB	80			
Human associated *Bacteroidales*	HF183	HF183F	ATCATGAGTTCACATGTCCG	1000	105	60	[2]
		BacR287R	CTTCCTCTCAGAACCCCTATCC	1000			
		BacP234P	FAM-CTAATGGAACGCATCCC-MGB	80			
Avian associated marker	GFD	GFD F	TCGGCTGAGCACTCTAGGG	100	123	57	[3]
		GFD R	GCGTCTCTTTGTACATCCCA	100			
Salmonella spp.	NA[a]	Sal F	GCTATTTTCGTCCGGCATGA	200	261	60	[4]
		Sal R	GCGACTATCAGGTTACCGTGGA	200			
		Sal Probe	FAM-TAGCCAGCGAGGTGAAAACGACAAAGG-TAMRA	250			
Arcobacter butzleri	*hsp60*	hsp60 F	CTCTTCATTAAAAGAGATGTTACCAATTTT	300	89	60	[5]
		hsp60 R	CACCATCTACATCTTCWGCAATAATTACT	300			
		hsp60 Probe	FAM-CTTCCTGATTGATTTACTGATT-NFQ-MGB	100			
Campylobacter jejuni	*mapA*	mapA F	CTGGTGGTTTTGAAGCAAAGATT	400	96	60	[6]
		mapA R	CAATACCAGTGTCTAAAGTGCGTTTAT	400			
		mapA Probe	FAM-TTGAATTCCAACATCGCTAATGTATAAAAGCCCTTT-TAMRA	80			
STEC	*STX2*	Stx2 F	CAGGCAGATACAGAGAGAATTTCG	200	68	61	[7]
		Stx2 R	CCGGCGTCATCGTATACACA	200			
		Stx2 Probe	VIC-ACTGTCTGAAACTGCTC-MGB	160			
Shigella sp.	*ipaH*	ipaH F	CTTGACCGCCTTTCCGATA	200	117	64	[8]
		ipaH R	AGCGAAAGACTGCTGTCGAAG	200			

		ipaH Probe	CY3-AACAGGTCGCTGCATGGCTGGAA-BHQ1	160			
		Entero F1A	AGAAATTCCAAACGAACTTG	200			
Enterococcus spp.	*ENT1A*	Entero R1	CAGTGCTCTACCTCCATCATT	200	92	60	[9]
		Entero Probe	FAM-TGGTTCTCTCCGAAATAGCTTTAGGGCTA-TAMRA	80			

[a] NA, not available.

Table S4. The limit of detection (LOD), limit of quantification (LOQ) and final assessment of qPCR results for each fecal marker and pathogen assays.

**Assay**	**Compiled Slope**	**Compiled Y-intercept**	**Compiled R^2 value**	**Compiled Efficiency (%)**	**LOQ (cp/µl)**	**LOD (cp/µl)**
BacUni	-3.076	39.777	0.999	111.386	100[a]	-
HF183 Taqman	-3.218	38.449	0.996	104.509	10 [a]	-
GFD	-3.658	36.997	0.995	87.651	10 [a]	-
Enterococcus spp. (*ENT1A*)	-3.563	41.004	0.999	90.847	10 [b]	3 [b]
Arcobacter butzleri (hsp60)	-3.3308	42.647	0.998	100.601	10 [b]	3 [b]
Campylobacter (*mapA*)	-3.408	39.608	0.998	96.541	10 [b]	3 [b]
Shigella spp. (*ipaH*)	-3.249	37.779	0.988	103.14	10 [b]	3 [b]
STEC (*stx2*)	-3.155	38.84	0.999	107.447	10 [b]	3 [b]
Salmonella spp.	-3.512	40.579	0.993	92.633	10 [b]	3 [b]

[a] Based on MST (Microbial source tracing) validation study in our group (Vadde et al. 2019) [10].

[b] Based on Oster et al. 2014.

Table S5. Two-way ANOVA of physico-chemical and microbiological parameters and abundance of fecal markers and pathogenic bacteria for water samples.

Source	df	SS	MS	F	P
**Water Temperature (WT)**					
A. Urbanization	2	0.026	0.013	9.099	**0.001**
B. Season	1	6.290	6.290	4406.812	**0.000**
A×B	2	0.009	0.005	3.170	0.056
**pH**					
A. Urbanization	2	0.002	0.001	6.074	**0.006**
B. Season	1	0.000	0.000	1.907	0.178
A×B	2	3.33E-05	1.67E-05	0.117	0.890
**Conductivity (EC)**					
A. Urbanization	2	0.097	0.049	5.050	**0.013**
B. Season	1	0.155	0.155	16.104	**0.000**
A×B	2	0.003	0.001	0.151	0.860
**Total Nitrogen (TN)**					
A. Urbanization	2	0.729	0.364	9.671	**0.001**
B. Season	1	0.177	0.177	4.691	**0.038**
A×B	2	0.123	0.061	1.632	0.212
**Total Phosphorus (TP)**					
A. Urbanization	2	3.216	1.608	19.592	**0.000**
B. Season	1	0.234	0.234	2.852	0.102
A×B	2	0.093	0.047	0.569	0.572
**Nitrate (NO_3)-N**					
A. Urbanization	2	0.024	0.012	0.205	0.816
B. Season	1	3.461	3.461	58.892	**0.000**
A×B	2	0.583	0.292	4.964	0.014
**Nitrite (NO_2)-N**					
A. Urbanization	2	0.156	0.078	0.887	0.422
B. Season	1	2.419	2.419	27.512	**0.000**
A×B	2	0.203	0.101	1.154	0.329
**Phosphate (PO_4)-P**					
A. Urbanization	2	7.207	3.604	69.510	**0.000**
B. Season	1	0.601	0.601	11.599	**0.002**
A×B	2	0.012	0.006	0.117	0.890
**Ammonia (NH_4)-N**					
A. Urbanization	2	3.917	1.958	12.379	**0.000**
B. Season	1	0.348	0.348	2.200	0.148
A×B	2	0.638	0.319	2.017	0.151
**Total Organic Carbon (TOC)**					
A. Urbanization	2	0.017	0.009	0.067	0.936
B. Season	1	0.014	0.014	0.107	0.745
A×B	2	0.037	0.018	0.144	0.867

**Chlorophyll *a***					
A. Urbanization	2	0.427	0.213	1.156	0.329
B. Season	1	3.097	3.097	16.775	**0.000**
A×B	2	0.293	0.147	0.795	0.461
**Total Viable Count (TVC)**					
A. Urbanization	2	2.553	1.276	3.612	**0.040**
B. Season	1	1.418	1.418	4.013	0.055
A×B	2	0.302	0.151	0.427	0.656
**Total Coliforms (TC)**					
A. Urbanization	2	3.938	1.969	8.519	**0.006**
B. Season	1	0.037	0.037	0.160	0.696
A×B	2	1.912	0.956	4.136	0.046
**Thermotolerant Coliforms/ Fecal Coliforms (TTC)**					
A. Urbanization	2	80422.33	40211.16	4.444	**0.036**
B. Season	1	53355.55	53355.55	5.897	**0.032**
A×B	2	22770.11	11385.05	1.258	0.319
**Bac Uni**					
A. Urbanization†	2	15.964	7.982	5.752	**0.008**
B. Season‡	1	0.261	0.261	0.188	0.668
A×B	2	0.085	0.042	0.031	0.970
**HF183**					
A. Urbanization	2	16.134	8.067	7.930	**0.002**
B. Season	1	2.989	2.989	2.938	0.097
A×B	2	0.044	0.022	0.021	0.979
***Enterococcus* spp.**					
A. Urbanization†	2	7.653	3.826	3.792	**0.034**
B. Season‡	1	1.098	1.098	1.088	0.305
A×B	2	0.061	0.030	0.030	0.970
Arcobacter butzleri					
A. Urbanization†	2	1.718	0.859	4.518	**0.019**
B. Season‡	1	80.149	80.149	421.466	**0.000**
A×B	2	2.396	1.198	6.299	0.005

† Urbanization: High vs. Medium vs. Low; ‡ Season: Winter vs. Summer;

df: degree of freedom; SS: Sum Square; MS: Mean Square

Table S6. Urbanization variation of physico-chemical and microbiological parameters and abundance of fecal markers and pathogenic bacteria for water samples.

Urbanization	Mean Difference	Std. Error	Sig. (P)	95% Confidence Interval	
				Lower Bound	Upper Bound
**WT**					
High vs. Medium	0.038	0.011	**0.002**	0.016	0.060
High vs. Low	-0.004	0.011	0.704	-0.026	0.018
Medium vs. Low	-0.042	0.011	**0.001**	-0.064	-0.020
**pH**					
High vs. Medium	-0.009	0.003	**0.019**	-0.016	-0.001
High vs. Low	-0.012	0.003	**0.002**	-0.019	-0.005
Medium vs. Low	-0.003	0.003	0.380	-0.010	0.004
**EC**					
High vs. Medium	0.001	0.028	0.974	-0.057	0.059
High vs. Low	0.078	0.028	**0.010**	0.021	0.136
Medium vs. Low	0.078	0.028	**0.010**	0.020	0.135
**TN**					
High vs. Medium	0.144	0.056	**0.016**	0.029	0.258
High vs. Low	0.245	0.056	**0.000**	0.131	0.360
Medium vs. Low	0.101	0.056	0.080	-0.013	0.216
**TP**					
High vs. Medium	0.349	0.083	**0.000**	0.180	0.518
High vs. Low	0.506	0.083	**0.000**	0.337	0.675
Medium vs. Low	0.157	0.083	0.067	-0.012	0.326
**NO_3-N**					
High vs. Medium	-0.032	0.070	0.651	-0.175	0.111
High vs. Low	0.011	0.070	0.874	-0.132	0.154
Medium vs. Low	0.043	0.070	0.542	-0.100	0.186
**NO_2-N**					
High vs. Medium	0.037	0.086	0.673	-0.138	0.211
High vs. Low	0.112	0.086	0.201	-0.063	0.287
Medium vs. Low	0.075	0.086	0.386	-0.100	0.250
**PO_4-P**					
High vs. Medium	0.484	0.066	**0.000**	0.350	0.619
High vs. Low	0.766	0.066	**0.000**	0.632	0.900
Medium vs. Low	0.282	0.066	**0.000**	0.147	0.416
**NH_4-N**					
High vs. Medium	0.375	0.115	**0.003**	0.140	0.609
High vs. Low	0.561	0.115	**0.000**	0.326	0.795
Medium vs. Low	0.186	0.115	0.116	-0.049	0.420
**TOC**					
High vs. Medium	-0.023	0.104	0.823	-0.235	0.188
High vs. Low	-0.037	0.104	0.720	-0.249	0.174

Medium vs. Low	-0.014	0.104	0.893	-0.225	0.197
**Chl *a***					
High vs. Medium	0.168	0.127	0.197	-0.092	0.428
High vs. Low	0.162	0.124	0.203	-0.092	0.415
Medium vs. Low	-0.006	0.127	0.962	-0.266	0.254
**TVC**					
High vs. Medium	0.497	0.249	0.055	-0.011	1.006
High vs. Low	0.617	0.243	**0.017**	0.121	1.114
Medium vs. Low	0.120	0.249	0.633	-0.388	0.629
**TC**					
High vs. Medium	0.571	0.278	0.064	-0.039	1.182
High vs. Low	1.215	0.294	**0.002**	0.567	1.863
Medium vs. Low	0.643	0.294	0.051	-0.005	1.291
**TTC**					
High vs. Medium	110.667	54.919	0.067	-8.991	230.324
High vs. Low	159.833	54.919	**0.013**	40.176	279.491
Medium vs. Low	49.167	54.919	0.388	-70.491	168.824
**Bac Uni**					
High vs. Medium	1.081	0.481	**0.032**	0.099	2.063
High vs. Low	1.598	0.481	**0.002**	0.616	2.581
Medium vs. Low	0.518	0.481	0.290	-0.464	1.500
**HF183**					
High vs. Medium	1.249	0.412	**0.005**	0.408	2.090
High vs. Low	1.545	0.412	**0.001**	0.704	2.386
Medium vs. Low	0.296	0.412	0.477	-0.545	1.137
Enterococcus spp.					
High vs. Medium	0.930	0.410	**0.031**	0.092	1.767
High vs. Low	1.020	0.410	**0.019**	0.182	1.857
Medium vs. Low	0.090	0.410	0.828	-0.748	0.928
Arcobacter butzleri					
High vs. Medium	0.258	0.178	0.157	-0.105	0.622
High vs. Low	0.535	0.178	**0.005**	0.171	0.899
Medium vs. Low	0.277	0.178	0.130	-0.087	0.640

Table S7. The median and 95th percentile data for TVC, TC and TTC for each urbanization gradient (High, Medium, Low) in Suzhou and Huangshan.

		TVC		TC		TTC	
		Median	95th percentile	Median	95th percentile	Median	95th percentile
Winter	H	40633	56283	9267	9927	95	118
	M	14367	43250	6667	8107	80	85
	L	9400	29383	67	787	0	21
Summer	H	42567	47867	8400	20730	230	455
	M	23000	47174	1400	2300	78	236
	L	21300	66167	500	3560	18	212
	Huangshan	22467	29847	178	858	3	16

Table S8A. Concentration of pathogenic bacterial genes (*Enterococcus* spp.) in water samples collected from Suzhou canals and streams in Huangshan.

	Enterococcus spp. Water (log_{10} gene copies/100mL)			
Location	**Winter 2015**	**Summer 2015**	**Winter 2016**	**Summer 2016**
1-1	**3.67**	**5.85**	**7.76**	**4.98**
1-2	**5.23**	**5.79**	**6.97**	**5.20**
1-3	**4.25**	**6.09**	**6.51**	**4.99**
2-1	**3.37**	**4.75**	**5.83**	**4.12**
2-2	**3.65**	**5.06**	**6.53**	**4.30**
2-3	**3.61**	**4.55**	**6.15**	**4.23**
3-1	**4.38**	**4.73**	**6.33**	**4.87**
3-2	**4.01**	**4.65**	**4.73**	**4.47**
3-3	**4.37**	**4.03**	**5.05**	**3.43**
H-1				**5.17**
H-2				**5.16**
H-3				**4.76**

Table S8B. Concentration of pathogenic bacterial genes (*Arcobacter butzleri*) in water samples collected from Suzhou canals and streams in Huangshan.

	Arcobacter butzleri Water (log_{10} gene copies/100mL)			
Location	**Winter 2015**	**Summer 2015**	**Winter 2016**	**Summer 2016**
1-1	DNQ	**5.97**	N.D	**4.82**
1-2	N.D	**5.03**	N.D	**4.89**
1-3	DNQ	**6.21**	N.D	**4.79**
2-1	DNQ	**4.34**	N.D	**4.54**
2-2	DNQ	**4.94**	N.D	**4.64**
2-3	**2.92**	**4.36**	N.D	**4.33**
3-1	DNQ	**4.24**	N.D	**5.04**
3-2	DNQ	**3.88**	N.D	**4.42**
3-3	DNQ	**3.36**	N.D	**4.05**
H-1				**4.25**
H-2				**3.80**
H-3				**3.69**

Table S8C. Concentration of pathogenic bacterial genes (*Shigella*) in water samples collected from Suzhou canals and streams in Huangshan.

Shigella				
	**Water (log_{10} gene copies/100mL)**			
Location	**Winter 2015**	**Summer 2015**	**Winter 2016**	**Summer 2016**
1-1	N.D	N.D	N.D	N.D
1-2	DNQ	DNQ	N.D	**2.81**
1-3	N.D	DNQ	N.D	**2.79**
2-1	N.D	N.D	N.D	DNQ
2-2	DNQ	N.D	N.D	**2.63**
2-3	N.D	N.D	**2.52**	DNQ
3-1	N.D	N.D	N.D	DNQ
3-2	N.D	DNQ	N.D	N.D
3-3	N.D	N.D	**2.31**	N.D
H-1				N.D
H-2				DNQ
H-3				N.D

Table S8D. Concentration of pathogenic bacterial genes (*Campylobacter jejuni*) in water samples collected from Suzhou canals and streams in Huangshan.

Campylobacter jejuni				
	**Water (log_{10} gene copies/100mL)**			
Location	**Winter 2015**	**Summer 2015**	**Winter 2016**	**Summer 2016**
1-1	N.D	N.D	N.D	N.D
1-2	**3.65**	N.D	N.D	N.D
1-3	N.D	N.D	N.D	N.D
2-1	N.D	N.D	**2.31**	N.D
2-2	N.D	N.D	**2.35**	N.D
2-3	N.D	N.D	N.D	N.D
3-1	N.D	N.D	N.D	N.D
3-2	N.D	N.D	DNQ	N.D
3-3	N.D	N.D	N.D	N.D
H-1				N.D
H-2				N.D
H-3				N.D

Table S8E. Concentration of pathogenic bacterial genes (*Salmonella* spp.) in water samples collected from Suzhou canals and streams in Huangshan.

Salmonella spp. Water (log_{10} gene copies/100mL)				
Location	**Winter 2015**	**Summer 2015**	**Winter 2016**	**Summer 2016**
1-1	N.D	N.D	N.D	N.D
1-2	N.D	N.D	N.D	N.D
1-3	N.D	**3.17**	N.D	N.D
2-1	N.D	N.D	DNQ	DNQ
2-2	N.D	N.D	N.D	N.D
2-3	N.D	N.D	N.D	N.D
3-1	N.D	N.D	N.D	N.D
3-2	N.D	N.D	**3.16**	N.D
3-3	N.D	N.D	N.D	N.D
H-1				N.D
H-2				N.D
H-3				N.D

Table S8F. Concentration of pathogenic bacterial genes (STEC) in water samples collected from Suzhou canals and streams in Huangshan.

STEC Water (log_{10} gene copies/100mL)				
Location	**Winter 2015**	**Summer 2015**	**Winter 2016**	**Summer 2016**
1-1	N.D	N.D	N.D	**3.23**
1-2	**2.92**	N.D	**2.88**	**3.44**
1-3	N.D	N.D	**3.05**	**3.36**
2-1	N.D	N.D	N.D	N.D
2-2	N.D	N.D	**2.91**	**3.02**
2-3	N.D	N.D	**2.87**	N.D
3-1	N.D	N.D	**3.19**	**3.28**
3-2	N.D	N.D	**2.96**	**3.14**
3-3	N.D	N.D	**2.89**	N.D
H-1				**2.86**
H-2				**2.88**
H-3				N.D

References

1. Kildare, B.J., et al., *16S rRNA-based assays for quantitative detection of universal, human-, cow-, and dog-specific fecal Bacteroidales: a Bayesian approach.* Water Res, 2007. **41**(16): p. 3701-15.
2. Green, H.C., et al., *Improved HF183 quantitative real-time PCR assay for characterization of human fecal pollution in ambient surface water samples.* Appl Environ Microbiol, 2014. **80**(10): p. 3086-94.
3. Green, H.C., et al., *Genetic markers for rapid PCR-based identification of gull, Canada goose, duck, and chicken fecal contamination in water.* Appl Environ Microbiol, 2012. **78**(2): p. 503-10.
4. Wang, L., Y. Li, and A. Mustapha, *Rapid and Simultaneous Quantitation of Escherichia coli O157:H7, Salmonella, and Shigella in Ground Beef by Multiplex Real-Time PCR and Immunomagnetic Separation.* Journal of Food Protection, 2007. **70**(6): p. 1366-1372.
5. de Boer, R.F., et al., *Detection of Campylobacter species and Arcobacter butzleri in stool samples by use of real-time multiplex PCR.* J Clin Microbiol, 2013. **51**(1): p. 253-9.
6. Best, E.L., et al., *Applicability of a rapid duplex real-time PCR assay for speciation ofCampylobacter jejuniandCampylobacter colidirectly from culture plates.* FEMS Microbiology Letters, 2003. **229**(2): p. 237-241.
7. Beutin, L., et al., *Evaluation of major types of Shiga toxin 2E-producing Escherichia coli bacteria present in food, pigs, and the environment as potential pathogens for humans.* Appl Environ Microbiol, 2008. **74**(15): p. 4806-16.
8. Ma, K., et al., *Rapid and simultaneous detection of Salmonella, Shigella, and Staphylococcus aureus in fresh pork using a multiplex real-time PCR assay based on immunomagnetic separation.* Food Control, 2014. **42**: p. 87-93.
9. Xue, J., et al., *Assessment of fecal pollution in Lake Pontchartrain, Louisiana.* Mar Pollut Bull, 2018. **129**(2): p. 655-663.
10. Vadde, K.K., et al., *Quantification of Microbial Source Tracking and Pathogenic Bacterial Markers in Water and Sediments of Tiaoxi River (Taihu Watershed).* Frontiers in Microbiology, 2019. **10**.
